# Effects of Recreational Boats on Harbour Porpoise Swimming Speed and Surfacing Interval Investigated by Two Synchronised UAVs

**DOI:** 10.1002/ece3.73165

**Published:** 2026-03-02

**Authors:** J. Till, V. Palmqvist, E. N. Wilk, P. Carlsson, J. Stedt

**Affiliations:** ^1^ Department of Biology, Functional Ecology Lund University Lund Sweden

**Keywords:** anthropogenic impact, behavioural effect, Cetacea, drone, noise, *Phocoena phocoena*

## Abstract

Cetaceans are negatively affected by anthropogenic activities, including acoustic and physical disturbance from boat traffic. Behavioural responses to such disturbances are context‐dependent, and site‐specific insights are needed for effective local management plans. In this study, the impact of speed and proximity of recreational boats on the swimming speed and surfacing interval of one of the most common coastal cetacean species, the harbour porpoise ( *Phocoena phocoena* ), is investigated using data collected by unmanned aerial vehicles (UAVs) within a key habitat for the vulnerable Belt Sea population. In August 2024, two UAVs were flown simultaneously on predefined routes within the area. One UAV searched for and followed detected porpoises, while the other monitored recreational boats. All data was captured as UAV video and used to determine surfacing intervals of individual porpoises, measure speed of porpoises and boats and calculate the closest distance between porpoises and boats for each simultaneous sighting. A total of 91 synchronous flights were conducted, resulting in 28 porpoise observational events. GLM analyses showed that an interaction between mean boat speed and distance to the boat influenced the mean speed of the porpoises. In the presence of boats with high mean speeds, porpoises at close range reduced their swimming speed, while porpoises at greater distances instead increased their swimming speed. Also, porpoise surfacing intervals decreased (i.e., porpoises surfaced more frequently) with decreasing distance to boats. This study demonstrates that recreational boats influence the behaviour of porpoises, which could lead to potential negative individual‐ and population‐level effects.

## Graphical abstract

This study examines the impact of speed and proximity of recreational boats on the swimming speed and surfacing interval of one of the most common coastal cetacean species, the harbour porpoise ( *Phocoena phocoena* ), using data collected by unmanned aerial vehicles (UAVs) in a key habitat for the vulnerable Belt Sea population. The analysis showed that the mean swimming speed of the harbour porpoises was influenced by the interaction of mean boat speed and distance to the boat, while the surfacing interval decreased with decreasing distance to the boats. This study demonstrates that recreational boats influence the behaviour of harbour porpoises, with potential negative individual‐ and population‐level effects.

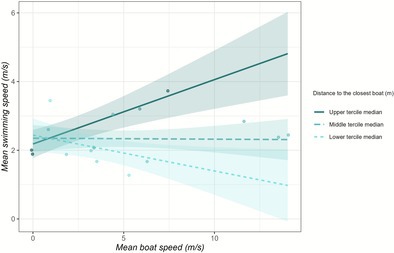

## 1 Introduction

Coastal areas are of great importance as a habitat for many cetaceans, partly due to their function as feeding grounds and nurseries (Awbery et al. 2019; Charles et al. 2025; Derville et al. 2019; Garaffo et al. 2011; Tubbs et al. 2020; White et al. 2023). These areas are, however, also intensively used for human activities, ranging from fishing operations (Izquierdo‐Serrano et al. 2022) to underwater construction (Chan and Karczmarski 2024) and boat and shipping traffic (Cezimbra et al. 2025; Pratezi and Rollo Jr. 2024), exposing cetacean species to strong negative anthropogenic impacts (Bearzi et al. 2009; Haviland‐Howell et al. 2007; Wilson et al. 2022).

The harbour porpoise ( *Phocoena phocoena* ; hereafter porpoise) is a small odontocete commonly found in marine coastal waters in the northern hemisphere (Jefferson 2008). Previous studies have identified a multitude of conflicts between porpoise habitat use and anthropogenic utilisation of inshore areas (Berggren et al. 2002; Dähne et al. 2013; Frankish et al. 2023; Hao et al. 2024; Oakley et al. 2017; Owen, Carlström, et al. 2024; Tougaard et al. 2009; Wisniewska et al. 2018), including conflicts related to boat and ship traffic (Bas et al. 2017; Frankish et al. 2023; Hao and Nabe‐Nielsen 2023; Hermannsen et al. 2014; Wisniewska et al. 2018). The physical and acoustic presence of various sizes and types of boats has previously been shown to have the potential to induce a wide range of behavioural responses in porpoises, such as explosive surfacing (Dyndo et al. 2015), a reduction of surface feeding and foraging activity (Bas et al. 2017; Wisniewska et al. 2018), a greater likelihood of switching between behavioural states (Bas et al. 2017), diving away (Frankish et al. 2023; Oakley et al. 2017), directional changes (Frankish et al. 2023; Hao et al. 2024), increased swimming speed (Hao et al. 2024) and possible abandonment of an area (Frankish et al. 2023). Given that porpoises spend the majority of their time in nearshore areas, which are often bordering densely human‐populated areas on land, recreational boat traffic and associated underwater noise are a real concern. Especially as seasonal increases in recreational boat traffic overlap with the reproductive and mating season of porpoises (IAMMWG et al. 2015; Lockyer and Kinze 2003). In fact, compared to commercial shipping, recreational vessels are expected to cause at least as much and possibly more underwater disturbance in these important coastal habitats, as larger vessels and ships mostly travel in offshore shipping lanes (Hermannsen et al. 2019). Nonetheless, the impact of recreational boats on porpoises is largely understudied, and information is lacking regarding when fitness impacts begin to occur (Tougaard et al. 2015).

Based on current knowledge, the response of porpoises to ships and boats seems to be influenced by a range of factors linked to the physical presence of the vessels, such as approach speed and distance (Bas et al. 2017; Hao et al. 2024; Oakley et al. 2017), as well as by acoustic disturbances that exceed a behaviour‐triggering threshold (Dyndo et al. 2015; Frankish et al. 2023; Tougaard et al. 2015; Wisniewska et al. 2018). In addition, the behavioural context, e.g., mating or swimming with a calf, as well as the environmental context, e.g., background noise, can be important factors influencing the response (Dyndo et al. 2015; Hao et al. 2024; Kastelein et al. 2011).

Understanding how recreational boats influence porpoises is crucial for sustainable use of relevant coastal habitats and for limiting associated conflicts between humans and porpoises. Like other odontocetes, porpoises depend on their biosonar for navigation, foraging and communication (Clausen et al. 2011; Sørensen et al. 2018; Verfuß et al. 2005; Villadsgaard et al. 2007). Their hearing range extends from approximately 13 kHz to 140 kHz, with their highest sensitivity at around 125 kHz (Kastelein et al. 2017, 2015). Boat noise often overlaps with this hearing range (Hermannsen et al. 2014; McKenna et al. 2012), thus, vessel and boat traffic may seriously affect their ability to use their primary sense (Erbe et al. 2019; Hermannsen et al. 2014). Given that porpoises have a very high metabolic rate and need to feed almost constantly (Kastelein et al. 2019; Rojano‐Doñate et al. 2018; Wisniewska et al. 2016), even subtle disturbances negatively affecting their time available for foraging or ability to locate and catch prey might have cumulatively large impacts on individual porpoises (Wisniewska et al. 2018) with possible population level effects. In the European Union (EU), the porpoise is strictly protected and listed in Annex II of the EU Habitats Directive (Council Directive 92/43/EEC 1992). To allow effective conservation of occurring vulnerable populations in European waters, increased knowledge regarding anthropogenic impacts on porpoises is needed (ASCOBANS 2012; HELCOM 2020). Also, fine‐scale local insights are essential as management plans need to be locally adapted (IAMMWG et al. 2015).

Unmanned aerial vehicles (UAVs; hereafter drones) are increasingly used for studies of porpoise behaviour (e.g., Brennecke et al. 2022; Hao et al. 2024; Stedt et al. 2024). Using drones, video data of undisturbed wild porpoises can be obtained, as the underwater noise disturbance from UAVs is negligible even when flying close to the surface (Christiansen et al. 2016). In addition, this method has the potential to allow longer and more detailed behavioural observations than is possible with conventional land‐ or boat‐based methods (Fettermann et al. 2022; Torres et al. 2018), which are often used to study interactions between porpoises and boats (Bas et al. 2017; Oakley et al. 2017). Moreover, since porpoises' reactions to ships are potentially subtle (Wisniewska et al. 2018), the high‐resolution drone‐based approach has the potential to reveal previously unrecognised patterns.

This study is based on focused data collection in a high‐density area and nursing ground for porpoises in Swedish waters, which is also heavily frequented by recreational boats. We use a novel drone‐based methodology, based on the simultaneous use of two drones for data collection, which allows detailed analysis of the reactions of porpoises to recreational boats. Thereby addressing some of the knowledge and methodological gaps outlined above. More specifically, the study aims to investigate behavioural changes of porpoises, i.e., surfacing interval and swimming speed, in relation to the nearest recreational boat and its travel behaviour. Focusing on these variables allows for an assessment of the disturbance caused by recreational boats, as changes in swimming speed and diving behaviour are known avoidance strategies (Frankish et al. 2023; Hao et al. 2024; Oakley et al. 2017), while also allowing inferences to be made about potential changes in metabolic rate (e.g., Christiansen et al. 2014; Gallagher et al. 2018). We hypothesise that porpoises attempt to avoid boats by (1) increasing their maximum and (2) mean swimming speed, the closer and faster recreational boats pass them. Furthermore, we expect porpoises to (3) increase their mean surfacing interval the closer and faster recreational boats pass them, as they dive longer and deeper to move out of their way.

## 2 Methods

### 2.1 Study Area

Data were collected within the Kullaberg Natura 2000 site in the northern part of Öresund, south‐west Sweden, which is recognised as a key habitat and high‐density area for the vulnerable Belt Sea porpoise population (HELCOM 2013; Stedt et al. 2023; Sveegaard et al. 2015; Sveegaard, Teilmann, Tougaard, et al. 2011; Sveegaard, Teilmann, Berggren, et al. 2011; Teilmann et al. 2022, 2008). In May–September, this Natura 2000 site is heavily frequented by recreational boats, as illustrated by a previous study recording 261 recreational boats throughout 34 sampling days in the summer of 2023 (Hartmann et al. 2025). These vessels account for the majority of boat and ship traffic in the area. During this time, porpoise mothers with calves are repeatedly sighted in the waters around Kullaberg (Hartmann et al. 2025; J. Till pers. obs.), indicating its importance as a nursing ground. The area is thus highly suitable for studies dependent on opportunistic observations, allowing detailed investigations into the impact of recreational boats on porpoises.

### 2.2 Data Collection

Data collection was carried out in August 2024, as this time of year offered comparably stable weather conditions paired with high numbers of recreational boating activity (J. Till pers. obs.). Flights were conducted opportunistically when weather conditions permitted (wind < 9 m/s, visibility ≥ 3 km and no rain). During August, 180 individual drone flights (i.e., 90 pairs) were carried out synchronously throughout 13 days with favourable conditions by two drone pilots. The time of day for data collection ranged from 7:30 am to 9:30 pm local time. Despite the relatively close proximity of sampling days, independence of observations was assumed, as porpoises are unlikely to habituate to boating noise (Dyndo et al. 2015) and the high abundance of porpoises in the area (HELCOM 2013; Stedt et al. 2023; Sveegaard et al. 2015; Sveegaard, Teilmann, Tougaard, et al. 2011; Sveegaard, Teilmann, Berggren, et al. 2011; Teilmann et al. 2022, 2008) makes repeated recordings of the same individual unlikely.

**FIGURE 1:**
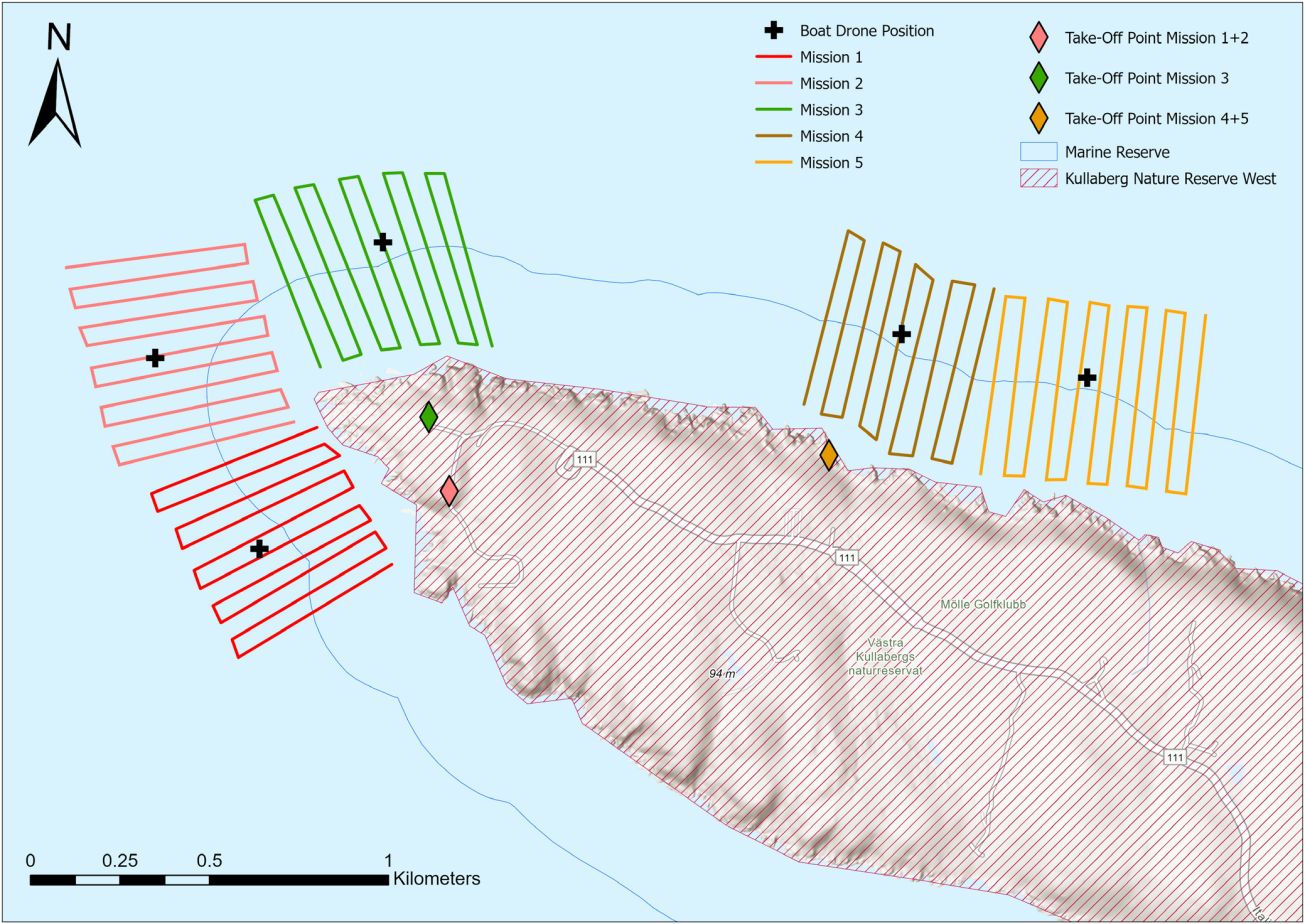
Study area and drone missions. The map shows the Kullaberg peninsula and the Kullaberg Natura 2000 site, which includes both the Kullaberg Nature Reserve West (magenta stripes) and the Marine Reserve (blue line). The take‐off points indicate where the drones were launched during data collection in August 2024. The long transects of each mission are approximately 500 m long, and the connecting shorter transects are approximately 50 m long. The missions stayed the same during the extent of the data collection and were flown at a height of 46 m above sea level. The black crosses indicate the position at which the DJI Phantom flew at 70 m above sea level and filmed the boats, whilst the DJI Mini flew the missions searching for and filming porpoises. Map lines delineate study areas and do not necessarily depict accepted national boundaries. Spatial reference system: WGS 1984 UTM Zone 33 N (EPSG:32633).

Drones used for data collection were the DJI Phantom 4 Pro (hereafter DJI Phantom) and the DJI Mini 3 (hereafter DJI Mini). The two drones collected data simultaneously; the DJI Mini tracked porpoises (resolution up to 4 K DCI, 3840 pixels × 2140 pixels, 30 frames/s) and the Phantom Pro tracked recreational boats (resolution up to 4 K DCI, 4096 pixels × 2140 pixels, 60 frames/s). Both drones were programmed based on the methodology in Hartmann et al. (2025), using the Litchi app (Version 1.0.0 Beta Build 350), to fly predefined routes (hereafter missions) within the study area (Figure 1). For each drone, a total of 5 missions split between three take‐off points were created (flight duration 15–20 min; Figure 1). Two take‐off points were located at the tip of the Kullaberg peninsula (56.300354° N, 12.451761° E; 56.302192° N, 12.450715° E) and one on the north side (56.301603° N, 12.468828° E). These take‐off points were chosen as they were easily accessible and located directly inland from areas with a known relatively high density of porpoises (Hartmann et al. 2025; Stedt et al. 2023) and boats (J. Till pers. obs.). On sampling days, a new mission was selected after each completed flight to ensure an effective search for porpoises in the study area.

Missions to record porpoise data were flown by the DJI Mini at an altitude of approximately 46 m above sea level during the initial search phase, with a maximum flight speed of 10 m/s and the camera pointing straight down at an angle of 90°. The flight altitude above sea level was calculated from the altitude above the take‐off point measured by the drone itself and the ground altitude of the take‐off point extracted from ‘Min Karta’ (Lantmäteriet 2024). Each mission consisted of between 9 to 11 almost parallel transect lines of approximately 500 m in length, which ran roughly against the depth gradient in the sea and were connected by transect lines of approximately 50 m in length (Figure 1). At this flight altitude, the visual overlap between the longer transect lines was approximately 22 m, as estimated by calculating the width of the area covered by the drone from its flight altitude and camera specs:

$$\text{width of covered area}=\frac{2*a*h*\tan\left(\frac{r}{2}\right)}{\sqrt{1+a^{2}}}$$

where *a* is the aspect ratio of the drone (1.9), *h* is the altitude of the drone during the mission (≈46 m) and *r* is the field of view of the drone camera in radians (≈1.466). There was no spatial overlap between individual missions, and each mission could be completed on a single battery charge. This design facilitated the efficient search for porpoises in an area. As soon as a porpoise came into view during a mission, the mission was stopped and the drone pilot reduced altitude to initiate a focal follow, i.e., a recorded video sequence in which a porpoise was followed by the drone. During these focal follows, the mean flight altitude was 32 m (min‐max range: 17 m–50 m), at which it is unlikely that the porpoises were disturbed or exhibited behavioural changes (Castro et al. 2021; Christiansen et al. 2016). The aim was to follow the porpoise for as long as possible. A focal follow was terminated if the porpoise disappeared for ≥ 10 s, e.g., because it dived or was lost in sight by the pilot. If the drone still had sufficient battery charge remaining at the end of a focal follow, the original mission was resumed. Data on the speed, acceleration and type of recreational boats in the survey area were recorded with the second drone, the DJI Phantom, at a height of 70 m above sea level. For each DJI Mini mission, a DJI Phantom mission was conducted in which the drone was flown to a stationary point near the centre of the survey area covered by the respective DJI Mini mission (Figure 1). From the centred position, the pilot panned the camera between all the boats in and around the survey area covered by the DJI Mini. The aim was to have surrounding boats in frame approximately every 10 s to allow interpolation of their travel path between known positions during times of simultaneous porpoise focal follow.

### 2.3 Data Extraction

From recorded videos, positions in latitude and longitude, as well as exact timestamps of observed boats and porpoises, were extracted in one‐second intervals using Drone Video Measure Version 1.1.1 (Egemose 2021; hereafter DVM). Further, a timestamp was obtained for all surface breaks of porpoises.

To assess how precisely annotations could be placed on boats, a trial data extraction was performed before the main analysis. These trials showed that boat positions further than 1 km away from the DJI Phantom could not be reliably used for calculations of boat driving speed and acceleration, as sufficiently accurate placement of annotations on the vessels was not possible due to limitations in video quality. Consequently, such calculations would likely have produced unrealistic values. However, the positions of these boats were considered precise enough to be used to estimate the distance to the porpoises, as this variable did not require calculations between successive positions. For boats between 315 m and 1025 m from the DJI Phantom flown at 70 m above the sea, the average accuracy of the estimated positions was 56 ± 37 m (mean ±1 standard deviation; Table S1).

A previous study by Brennecke et al. (2022) found a positional error of 2.4 ± 1.5 m (mean ±1 standard deviation) for the DJI Mini when flown at an altitude of 30 m. Given that the recording and extraction setup of this study largely resembled the setup of this previous study, we adopted their reported positional accuracy for our focal follow porpoise detections.

### 2.4 Data Processing

**FIGURE 2:**
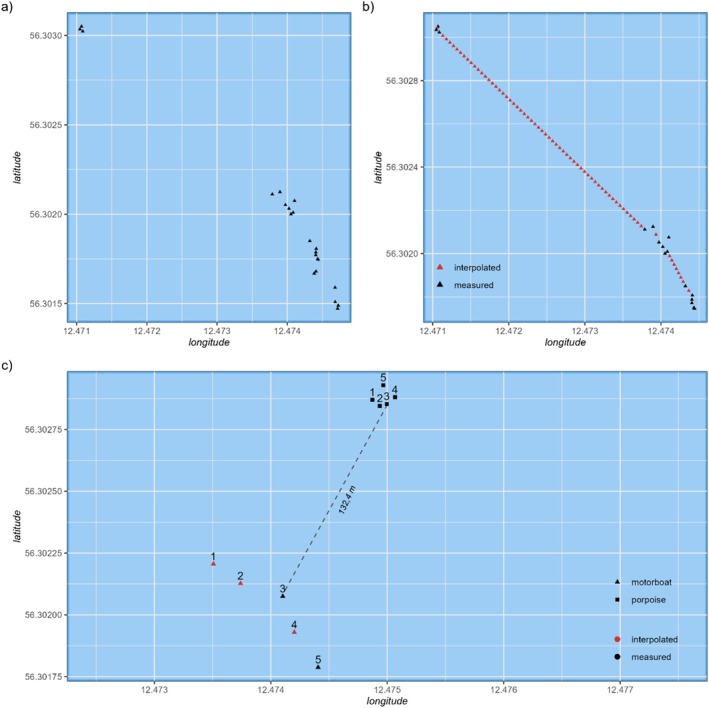
Visualisation of the movement paths, interpolation and distance calculation. The two upper diagrams show only the path travelled by the boat, with (a) the positions extracted from the video footage and (b) adding the positions estimated by interpolation in red. In the lower diagram (c), the positions of the boat (triangles) are combined with the positions (squares) of the porpoise, which was simultaneously filmed by the DJI Mini. The numbers indicate the chronological order of the positions, with one being the first and five being the last position. Boat and porpoise positions with matching numbers represent the simultaneity of the positions. The red points symbolise those that were estimated by interpolation. The dashed line shows the closest distance between the porpoise and the boat of 132.4 m. The lower graph (c) only shows points at 5‐s intervals to improve visualisation.

In some cases, the exact positions of boats could not be continuously extracted due to camera panning. Such missing positions during porpoise focal follows were interpolated in one‐second intervals using the *zoo*‐package for R (Zeileis and Grothendieck 2005). The *sf*‐package for R (Pebesma 2018; Pebesma and Bivand 2023) was then used to calculate exact distances between the temporally matched individual boat locations and corresponding porpoise locations at approximately every second of the focal follow (Figure 2). For the analysis of the effects of boat distances on porpoise behaviour, the distance of the boat closest to the respective porpoise was used.

Speed and acceleration were only calculated for boats within a 1 km radius from the centred DJI Phantom position, and QGIS 3.36.2 was used to find all occasions when the closest boat was inside this threshold. Whenever this was the case, porpoise travel speed was calculated between consecutive porpoise positions. Data points where these calculations resulted in implausible porpoise speeds of more than 6.2 m/s (Gaskin et al. 1974; Hao et al. 2024; Leatherwood et al. 1982; Otani et al. 2000) were excluded from further analysis to avoid inclusion of potentially biased data presumably due to difficulties in accurately placing annotations during deeper dives. In total, 30 out of 656 values had to be excluded in accordance with this procedure. For each boat identified as being closest to the porpoise during a focal follow, it was assessed whether it was motorised (i.e., actively driving using a motor), and the speed between consecutive boat positions was also calculated. Again, some calculations gave unlikely high speeds of > 16.6 m/s between consecutive positions (Hao and Nabe‐Nielsen 2023), resulting in the exclusion of data points. However, only 2 out of 446 speed calculations were removed as the remaining values fell within the range reported for recreational boats in coastal waters of the Belt Sea (Hao and Nabe‐Nielsen 2023). In addition, one boat track was excluded due to implausible speed values in relation to the vessel type. Following the removal of data points with unlikely porpoise and boat speeds, the remaining data were considered reliable and suitable for continued data processing and analysis.

From the cleaned dataset with temporally paired observations of boats and porpoises at 1 s resolution, the mean and maximum speed of each porpoise were determined. The mean surfacing interval was calculated for each porpoise that surfaced ≥ 4 times. For the boat closest to the porpoise during the focal follow, the mean speed, maximum speed and maximum acceleration were calculated from the cleaned values of each vessel. For five porpoise‐boat pairs, the closest boat was filmed only shortly before and after the focal follow; therefore, for these five pairs, the positions of the closest boat were determined exclusively by interpolation, assuming a non‐accelerated linear movement. As a result of having to interpolate the positions for these pairs, only the mean speed was included in the following analysis. Further, during calculation, some of the closest boats yielded implausible maximum accelerations (> 15 m/s^2^), which led to the exclusion of these boat–porpoise pairs from the analysis.

### 2.5 Data Analysis

Generalised Linear Models (hereafter GLMs) with a Gaussian distribution were fitted in R to the drone data to investigate how the mean and maximum swimming speed, as well as the mean surfacing interval, of porpoises changed in relation to the distance to the closest boat, its maximum and minimum speed and its maximum acceleration. Given the small sample sizes, all explanatory variables were analysed individually to avoid overfitting of models. The only model with multiple explanatory variables was the one which examined the effect of the interaction between mean boat speed and distance to the closest boat on mean and maximum swimming speed (Tables 2, 3 and 5). This analysis was possible as the sample size for this interaction was considered sufficient. The interaction was further visually investigated using the *interactions* package in R (Long 2024) to create interaction plots that split the data into three equal‐sized groups and plotted the median distance to the closest boat as a line for each group, providing a biologically interpretable visualisation.

For all models, the assumptions of normality of the residuals were tested with the Shapiro–Wilk test, and the assumptions of homogeneity of variance with the studentised Breusch–Pagan test from the *lmtest* package for R (Zeileis and Hothorn 2002). The mean surfacing interval as a function of the distance to the closest boat required a log transformation of the response variable to fulfil the assumptions. The fit of the models was analysed using diagnostic plots and the corrected Akaike information criterion (hereafter AICc).

Figures of the best‐fitting models were created in R using the *ggplot2* package (Wickham 2016).

## 3 Results

### 3.1 Swimming Speed

**FIGURE 3:**
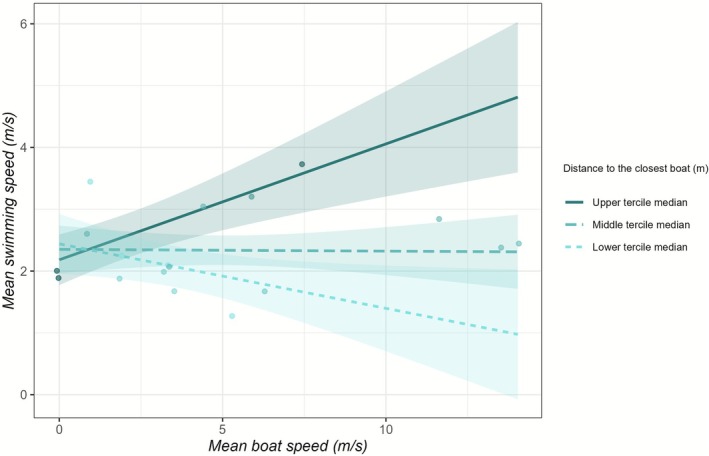
Effect of the interaction between the mean boat speed (m/s) and the distance to the closest boat (m) on the mean swimming speed of porpoises (m/s). The points in the plot are the individual data points, and the darker the colour, the further away the boats are from the porpoise. The lower, middle and upper terciles of the distances to the closest boat range from 121 m to 315 m, 351 m to 606 m and 627 m to 2318 m, respectively. The trend lines represent the effect of the mean boat speed on the mean swimming speed of the porpoises at the median of the respective tercile: 135 m, 564 m and 1368 m. The shading indicates the 95% confidence interval.

The data collection yielded a total of 28 cleaned focal follows with matching data from recreational boats, averaging at 29.7 s in length. Throughout the sampling period, no porpoises were detected or filmed in the absence of boats. Porpoise mean speed was 2.4 m/s, with a maximum speed of 6.2 m/s. In comparison, boats travelled at a mean speed of 4.6 m/s, with a maximum speed of 14.3 m/s. The highest recorded boat acceleration was 6.9 m/s^2^. On average, boats were located 1045 m (min‐max range: 121 m–2532 m) from the porpoises. Out of the 28 cleaned focal follows, 18 (64%; proportion motorised = 77.8%) could be used to model effects of the interaction between distance to the closest boat and mean boat speed on porpoise swimming speeds. For this subset, boats were on average 713 m (min‐max range: 121 m–2318 m) from the porpoises. The interaction model for porpoise mean swimming speed showed that it was influenced by the interaction between mean boat speed and distance to the closest boat (*p* < 0.01; Table 1; Figure 3). Additionally, mean boat speed had a significant negative effect on porpoise mean swimming speed (*p* < 0.05; Table 1); however, because it is involved in the interaction term, the interpretation of this main effect in isolation is statistically meaningless (Genç and Mendeş 2022). Visual investigation of the interaction using the exemplary values of the medians of terciles, each containing the same number of data points and defined by the distance to the closest boat, revealed that the distance to the closest boat influenced the direction of the porpoises' response to mean boat speed (Figure 3). For a median boat distance of 135 m for the lower tercile, porpoise mean swimming speed decreased as a function of increased boat speed. For the median value of 564 m of the middle tercile, mean boat speed did not affect porpoise swimming speed. However, for the median distance of 1368 m for the upper tercile, the relationship between porpoise swimming speed and boat speed was reversed compared to the lower tercile, with porpoise mean swimming speed increasing as a function of increased mean boat speed. Porpoise maximum swimming speed was not affected by the interaction between distance to the closest boat and mean boat speed (Table 2).

**TABLE 1:** Summary statistics of the Gaussian GLM predicting mean porpoise speed as a function of mean boat speed and distance to the closest boat, including their interaction. The model had 17 degrees of freedom, an adjusted *R* ^2^ of 0.494 and an AICc of 33.86.

Predictor	Estimate	Std. error	*t*‐value	*p*
Intercept	2.4690	0.2437	10.128	< 0.001***
Boat distance	−0.0002	0.0002	−1.063	0.306
Mean boat speed	−0.1363	0.0515	−2.646	0.019*
Boat distance*mean boat speed	0.0002	0.0001	3.984	0.001**

*Note:* Significance codes: ‘***’ 0.001, ‘**’ 0.01, ‘*’ 0.05.

**TABLE 2:** Candidate Gaussian GLMs for the maximum swimming speed of porpoises.

Response variable	Predictor	AICc	AICc null model
Maximum swimming speed	Mean boat speed * Distance to the closest boat	53.574	49.052
	Mean boat speed + Distance to the closest boat	55.011	49.052
	Distance to the closest boat	76.575	74.510
	Mean boat speed	51.966	49.052
	Maximum boat speed	41.335	39.042
	Maximum boat acceleration	40.173	39.042

*Note:* * indicates interactions between explanatory variables. The AICc (corrected Akaike information criterion) values of the null models differ due to the different sample sizes. The lower the AICc value, the better the model fit. If the null model had a lower AICc value than the corresponding model with a predictor, the predictor did not lead to an improvement in model fit.

When modelling effects of the individual predictors on the swimming speed of porpoises, all of the 28 focal follows were used for distance to the closest boat (100%; proportion motorised = 82.1%), 18 for mean boat speed (64%; proportion motorised = 77.8%) and 13 for maximum boat speed and maximum boat acceleration (46%; proportion motorised = 76.9%). None of these models and predictors could explain the variance in mean and maximum swimming speed, as none of the AICc values were lower than that of the null model (Tables 2 and 3).

**TABLE 3:** Candidate Gaussian GLMs for the mean swimming speed of porpoises.

Response variable	Predictor	AICc	AICc null model
Mean swimming speed	Mean boat speed * Distance to the closest boat	33.863	39.411
	Mean boat speed + Distance to the closest boat	43.583	39.411
	Distance to the closest boat	60.894	58.793
	Mean boat speed	41.325	39.411
	Maximum boat speed	33.358	31.550
	Maximum boat acceleration	34.683	31.550

*Note:* The * indicates interactions between explanatory variables. The AICc (corrected Akaike information criterion) values of the null models differ due to the different sample sizes. The lower the AICc value, the better the model fit. If the null model had a lower AICc value than the corresponding model with a predictor, the predictor did not lead to an improvement in model fit.

### 3.2 Surfacing Interval

**FIGURE 4:**
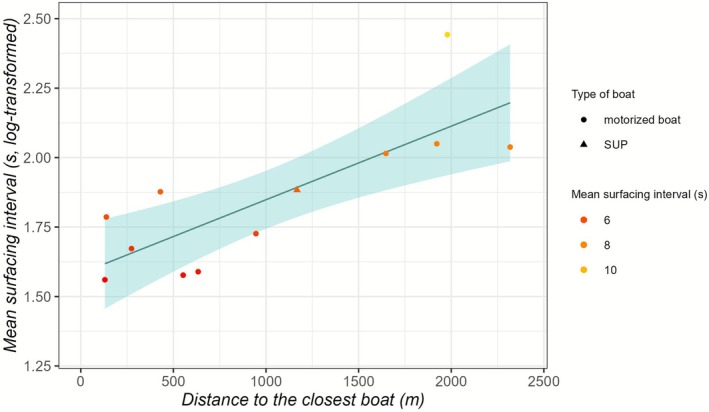
Effect of the distance to the closest vessel (m) on the mean surfacing interval in seconds (log‐transformed). The points of the scatter plot are the individual data points, the trend line is the fitted GLM with the 95% confidence interval in light blue. The shape of the points indicates the type of the boat, the colour the mean surfacing interval in seconds. The mean surfacing interval decreases significantly the closer the boats are (*p* < 0.01, adj. *R* ^2^ = 0.6).

Surfacing intervals for porpoises could be calculated in 12 out of the 28 focal follows with the corresponding boat data (43%; proportion motorised = 92%) and provided a mean of 6.6 s (min‐max range: 4.8 s–11.5 s). The mean distance to the closest boat for this subset of the data was 1012 m (min‐max range: 130 m–2318 m). When analysing the mean surfacing interval as a function of the distance to the closest boat, all 28 focal follows could be used. The porpoises significantly reduced their mean surfacing interval with decreased distance to the closest boat (*p* < 0.01; Table 4; Figure 4).

**TABLE 4:** Summary statistics of the Gaussian GLM predicting the mean surfacing interval as a function of distance to the closest boat. The model had 11 degrees of freedom, an adjusted *R* ^2^ of 0.6 and an AICc of −2.74.

Predictor	Estimate	Std. error	*t*‐value	*p*
Intercept	1.5830	0.0790	20.038	< 0.001***
Boat distance	0.0003	0.0001	4.212	0.002**

*Note:* Significance codes: ‘***’ 0.001, ‘**’ 0.01, ‘*’ 0.05.

For the mean boat speed, maximum boat speed and maximum boat acceleration, sample sizes of 7 (25%; proportion motorised = 100%), 6 (21%; proportion motorised = 100%) and 6 (21%; proportion motorised = 100%) out of the 28 focal follows were used, respectively. None of these explanatory variables could explain the mean surfacing interval, as the respective null models had a lower AICc (Table 5).

**TABLE 5:** Candidate Gaussian GLMs for the mean surfacing interval of porpoises.

Response variable	Predictor	AICc	AICc null model
log (Mean surfacing interval)	Distance to the closest boat	−2.744	5.835
	Mean boat speed	33.320	26.933
Mean surfacing interval	Maximum boat speed	29.728	19.971
	Maximum boat acceleration	29.967	19.971

*Note:* When modelling the mean surfacing interval as a function of the distance to the closest boat, a log transformation was necessary to fulfil the assumptions of the GLM. The AICc (corrected Akaike information criterion) values of the null models differ due to the different sample sizes. The lower the AICc value, the better the model fit. If the null model had a lower AICc value than the corresponding model with a predictor, the predictor did not lead to an improvement in model fit.

## 4 Discussion

This study demonstrates that recreational boats influence the behaviour of porpoises, but that the effects are complex and context dependent as they change intricately in response to varying boat characteristics. Using a novel UAV methodology utilising two UAVs for the collection of temporally linked boat‐porpoise data, we found that the interaction between mean boat speed and distance to the boat influenced the mean speed of porpoises. At close range, porpoises slowed down their swimming speed in response to boats with high mean speeds, but over longer distances, they increased their mean swimming speed. Additionally, porpoise surfacing intervals decreased as boats came closer. No effect of maximum boat speed on porpoise behaviour was observed. These responses indicate disturbance from recreational boat traffic that could potentially bring energetic or stress‐related consequences for porpoises. This study does not allow conclusions to be drawn with certainty as to whether physical presence or acoustic disturbance caused the observed behavioural changes; however, despite the lack of acoustic data, we discuss possible causes.

### 4.1 Swimming Speed Modification in Relation to Speed and Proximity to Boats

As expected, the mean porpoise swimming speed was influenced by the interaction between the mean boat speed and the distance to the closest boat. At short distances from boats, porpoises decreased their mean swimming speed in response to increased mean boat speeds. This is in direct contradiction to previous findings, which have found porpoises to increase their speed in response to boats (Hao et al. 2024; Wisniewska et al. 2018). In Hao et al. (2024), the speed at which the boats caused an increase in porpoise swimming speed was more than double the mean speed of the boats in this study, offering a potential explanation for the difference in results. The reduced mean swimming speed could be a behavioural response of porpoises to anthropogenic noise, as has been reported for other cetaceans (Johnston and Painter 2024; Weilgart 2007). This response might reflect masking of echolocation signals and communication by the underwater sounds emitted by recreational boats, especially as the mostly motorised boats recorded in this study likely transmit noise intensely at high speeds and over short distances, based on previous studies on the effect of recreational boats on the underwater soundscape (Hermannsen et al. 2019). Alternatively, the observed reduction in swimming speed could reflect a behavioural strategy by porpoises to minimise energy consumption and counteract reduced foraging due to boat disturbance (Bas et al. 2017; Wisniewska et al. 2018) or a stress‐induced increase in metabolic rate (Christiansen et al. 2014), as the overall metabolic costs of porpoises are expected to increase with increased swimming speed (Gallagher et al. 2018).

At medium range distances to the closest boat, porpoises did not change their mean swimming speed in relation to increased mean boat speeds. These results are similar to previous studies showing that porpoises' reactions to boats are most likely to occur at distances of 100 m to 300 m (Frankish et al. 2023; Hao et al. 2024; Oakley et al. 2017). Given this established spatial range where behavioural responses are probable to occur, our results with a documented increase in mean swimming speed as a function of mean boat speed at relatively large distances are surprising. Nevertheless, long‐range responses are not unprecedented, as porpoises have been documented to respond to ships at distances of up to 7 km (Wisniewska et al. 2018), and Frankish et al. (2023) reported a response probability of 5%–9% at distances of up to 2 km.

The sound received by the porpoise over longer distances from the predominantly motorised boats with increased mean driving speeds is likely less intense than at short distances (Hermannsen et al. 2019). This might result in distance‐dependent avoidance strategies. This study focused on investigating changes in swimming speed as a potential avoidance strategy. The lack of an observed response to increased mean boat speeds at medium distances does not necessarily mean that porpoises were undisturbed; instead, they might switch to an avoidance strategy which we were unable to detect using speed as a response variable. For example, porpoises have previously been reported to respond to and move away from boats by changing directions (Frankish et al. 2023; Hao et al. 2024). In our analysis focusing on speed, directional changes as a response to boats were not included, and it is possible that when boats with increased mean speeds were at medium distances, this was the strategy adopted by porpoises. It could be hypothesised that the increase in mean swimming speed in response to increased mean boat speeds at greater distances represents an avoidance attempt to escape an approaching boat, as indicated by previous studies (Christiansen et al. 2014; Hao et al. 2024; Sprogis et al. 2020; Wisniewska et al. 2018). This response might be adapted to the level of disturbance experienced through the interaction of individual boat characteristics and likely comes at an energetic cost for porpoises (Gallagher et al. 2018).

Our findings provide support that porpoises react more strongly to boats at higher mean speeds (Bas et al. 2017; Oakley et al. 2017) when modelled in interaction with the distance to the closest boats. This could be because faster motorboats are more likely to exceed the sound response thresholds identified in previous studies (Dyndo et al. 2015; Hermannsen et al. 2019; Tougaard et al. 2015; Wisniewska et al. 2018). Furthermore, the results show that the direction of the response to the mean speed of the boats depends on the distance to the porpoises, potentially due to differences in experienced noise levels (Hermannsen et al. 2019). This provides a new perspective on the previously documented increased likelihood of porpoises diving away (Frankish et al. 2023; Oakley et al. 2017), changing direction (Frankish et al. 2023), or reducing foraging (Bas et al. 2017) with decreased distance to boats. The significance of interactions between affecting factors is further emphasised, as the mean boat speed and the distance to the nearest boat had no effect when examined individually. It appears that porpoises' behavioural options and decisions are influenced more by the cumulative characteristics of the boat than by any single factor. These findings support the idea that multiple factors play a role in shaping porpoise responses (Dyndo et al. 2015; Hao et al. 2024). Because the reactions to mean boat speed were strongly dependent on the distance to the boat, and the direction of the response was contradictory at short versus long distances, a GLM investigating predictors individually might fail to detect significant relationships, as opposing effects can cancel each other out.

The mechanisms underlying the observed differences in swimming speed reactions of porpoises, and whether these are driven by the physical presence of the boats or acoustic disturbances, cannot be determined with certainty in this study, as this would require simultaneous quantitative data on the local underwater noise background. Studies combining acoustic and drone‐based methods would allow linking observed behavioural responses to acoustic data. Further investigations of the directions of porpoise movements in response to boats are likely to provide valuable insights.

Additionally, these results should be interpreted with caution, as the sample size was small and the model might overfit the data. To verify the trends described here, further investigations with a larger sample size are needed. Moreover, supplementing the drone with a theodolite could improve the accuracy of speed and distance calculations (Piwetz et al. 2018). However, data cleaning reduced the number of erroneous measurements. In addition, although control data collected in the complete absence of boats were not available due to the high frequency of boats, the range of boat distances and speeds experienced by porpoises is well represented in the dataset. We therefore argue that the observed responses reflect the porpoise behaviour across varying degrees of disturbance.

Unexpectedly, maximum boat speed did not affect the mean swimming speed of the porpoises, which may be due to the short duration of the maximum speed, as the disturbance duration can influence the detection threshold of porpoises and their reactivity (Kastelein et al. 2010; Tougaard et al. 2015). Nevertheless, it has been shown previously that porpoises react even to short and impulsive noise disturbances (Kastelein and Gransier 2013; Kastelein et al. 2011). In such situations, the response seems to be influenced by the surrounding broadband noise (Kastelein et al. 2011) which, given the lack of acoustic data collection in this current study, could introduce bias unaccounted for. Moreover, the maximum acceleration of the boat did not influence the mean porpoise swimming speed. This contrasts with earlier findings, in which boats moving at a constant speed, and therefore with minimal to no acceleration, elicited stronger behavioural responses in porpoises than those travelling at varying speeds (Oakley et al. 2017). However, the responses of porpoises to recreational boats may have a high degree of plasticity, potentially depending on, e.g., environmental context, porpoise group structure and behavioural state. Also, our analysis focused on the closest distance between boat and porpoise in each focal follow, rendering the exact distance between porpoise and boat during the boat's maximum speed and acceleration unknown. As a result, it is possible that we slightly underestimated the effects recreational boats have on porpoises. However, this influence is likely minimal given the relatively short average duration of the focal follows.

Unlike the mean swimming speed, the maximum swimming speed of porpoises was not influenced by the speed, proximity, or acceleration of the nearest boat. This could indicate a subtle rather than an intense behavioural response by porpoises to boat traffic. This does, however, not mean that the impact on porpoise behaviour is negligible, as moderate disturbances can potentially have a cumulatively large impact (New et al. 2014; Weilgart 2007). The lack of evidence that porpoises leave the area quickly, which could be indicated by high swimming speeds, provides support to the existing theory that they might be unwilling to leave a potentially valuable feeding ground despite disturbance, as they are so dependent on reliable access to prey (Frankish et al. 2023; Owen, Carlström, et al. 2024; Wisniewska et al. 2018). Future aerial surveys focusing on the swimming direction of porpoises in response to boats could provide reliable information on whether they are actively moving away from boats or adopt other avoidance strategies. Such studies should then also investigate whether porpoises that move away or avoid recreational boats in other ways return to their original position following the disturbance. This is important as several small deviations from the original position could lead to a gradual displacement from the area, as suggested by Frankish et al. (2023).

### 4.2 Surfacing Interval Decrease in Response to Boat Proximity

We expected porpoises to increase their mean surfacing interval, i.e., diving for longer time periods and to deeper depths, in response to closer proximity and increased driving speed of recreational boats, as a way to move out of their way.

Our results do, however, provide no support for this as the observed porpoises did not change their surfacing behaviour as assumed. Neither the maximum, the mean speed, nor the maximum acceleration of the boats could explain the observed variance. These results might be biased due to the small sample size used to analyse boat speed and acceleration. Nonetheless, they are partly consistent with recent findings, where porpoises did not change their breathing behaviour when exposed to boats travelling at 10 and 20 knots (Hao et al. 2024).

Contrary to what we expected, porpoises reduced their mean surfacing interval as recreational boats came closer. These results differ from previous findings, suggesting that boat proximity does not affect the surfacing rate (Hao et al. 2024). Furthermore, they deviate from studies showing that porpoises use diving as an avoidance strategy (Frankish et al. 2023; Oakley et al. 2017; Wisniewska et al. 2018). These contrasting results may indicate a context‐dependent response of porpoises, as has also been previously suggested by Hao et al. (2024) and Dyndo et al. (2015). In the relatively deep waters (approx. 30 m) of our study area, the mean surfacing interval might not be a good indicator of avoidance behaviour through diving, as individual porpoises cannot be continuously tracked during deep dives (> 5 m) and focal follows had to be terminated in such cases. As a result, this study is biased towards shallower dives during which individual porpoises could be successfully tracked throughout a dive. Consequently, the time during which surfacing behaviour could be assessed per focal follow was comparatively short, which may have led to an overestimation of the mean surfacing interval, as has been found for other cetaceans (Azizeh et al. 2021; Christiansen et al. 2025). Moreover, porpoises may surface more frequently because they are stressed by the presence of boats. Minke whales ( *Balaenoptera acutorostrata* ), for example, have been shown to reduce their surfacing intervals in the presence of boats due to a stress‐related increase in metabolic rate (Christiansen et al. 2014).

### 4.3 Possible Implications for Long‐Term Fitness and Conservation

Disturbances requiring animals to modify their behaviour and adopt avoidance strategies can be energetically costly and lead to forced transfer of energy investment from, e.g., foraging and reproduction (Bas et al. 2017; Gallagher et al. 2021; Hin et al. 2021, 2019; Meza et al. 2020; Symons et al. 2014). Additionally, the observed behavioural changes of porpoises in response to recreational boat traffic described in this study might indicate masking effects and stress. Given the constantly high foraging needs of porpoises, notably in reproducing females, these animals are expected to have a limited ability to compensate for such anthropogenic disturbance in their search for food (Wisniewska et al. 2016). Therefore, such disturbances could result in long‐term fitness consequences (Kastelein et al. 2019; Wisniewska et al. 2016), especially as the physical condition and energy balance of adult female porpoises directly affect their reproductive success (Gallagher et al. 2021). The co‐occurrence of porpoises and recreational boats in key habitats during the summer months is hence of specific concern, as female porpoises with young calves are likely to be particularly susceptible to disturbances. Furthermore, porpoises might have limited flexibility in finding and moving into suitable replacement areas for foraging or nursing if their primary areas become too busy with human activities.

Despite the inclusion of porpoises in Annex II of the EU Habitats Directive (Council Directive 92/43/EEC 1992) and the establishment of SACs (ASCOBANS 2012), there is a lack of implementation of management plans and concrete measures to avert threats (Carlén et al. 2021). The vulnerable Belt Sea population of porpoises (HELCOM 2013) is declining (Owen, Gilles, et al. 2024). While bycatch poses the primary threat, a comprehensive understanding of all potential risks to individual animals is critical for developing effective management plans (IAMMWG et al. 2015). This study contributes to a growing body of research that highlights the potential negative impacts of recreational boats on porpoises in nearshore coastal waters, which are crucial foraging and reproductive habitats for the species, thereby facilitating the design and implementation of targeted conservation actions.

## 5 Conclusions

This study adds to the small but growing number of studies investigating the effects of recreational boat traffic on the behaviour of harbour porpoises. Using a novel UAV‐based approach for data collection, we show that boat speed and distance to porpoises influence how porpoises react to this disturbance.

These results are important from a management perspective. They can serve as guidance for regional managers, not only for porpoises but also for other marine species with similar habitat preferences and behaviour. Finally, this study provides a new methodological approach for research and monitoring of cetacean behaviour and highlights how the methodology could be improved by combining it with, e.g., acoustic data.

## Author Contributions

**J. Till:** conceptualization (lead), data curation (lead), formal analysis (lead), investigation (equal), methodology (lead), visualization (lead), writing – original draft (lead), writing – review and editing (equal). **V. Palmqvist:** data curation (supporting), investigation (equal). **E. N. Wilk:** investigation (equal), visualization (supporting). **P. Carlsson:** supervision (supporting), writing – review and editing (equal). **J. Stedt:** conceptualization (supporting), funding acquisition (lead), methodology (supporting), project administration (lead), resources (lead), supervision (lead), writing – original draft (supporting), writing – review and editing (equal).

## Funding

This work was supported by the Gyllenstierna Krapperup´s Foundation, KR2024‐0081.

## Conflicts of Interest

The authors declare no conflicts of interest.

## Supporting information


**Table S1:** Error measurements for the DJI Phantom. The measurements were carried out at an altitude of around 70 m above sea level. The annotations were placed on targets with a known position, with which the accuracy of the extracted position was compared. The mean error is 56 m, and the standard deviation is 36 m.

## Data Availability

The raw data underpinning the conclusions and analyses of this study, as well as the R code used to calculate distances, swimming speeds, driving speeds and accelerations, are available on the open publishing data platform Dryad, DOI https://doi.org/10.5061/dryad.j0zpc86v7.
