# Supplementary material for: Effects of Recreational Boats on Harbour Porpoise Swimming Speed and Surfacing Interval Investigated by Two Synchronised UAVs
Source: Ecol Evol. 2026 Mar 2;16(3):e73165. doi: 10.1002/ece3.73165 (PMC12952998; doi:10.1002/ece3.73165)
Supplement: Supplementary file 1 — Table S1: Error measurements for the DJI Phantom. The measurements were carried out at an altitude of around 70 m above sea level. The annotations were placed on targets with a known position, with which the accuracy of the extracted position was compared. The mean error is 56 m, and the standard deviation is 36 m. [file ECE3-16-e73165-s001.docx]

**Appendix A**

**Table A1**. Error measurements for the DJI Phantom. The measurements were carried out at an altitude of around 70 m above sea level. The annotations were placed on targets with a known position, with which the accuracy of the extracted position was compared. The mean error is 56 m, and the standard deviation is 36 m.

| **Distance Between Drone and Target (m)** | **Measurement Error (m)** |
| --- | --- |
| 315 | 2 |
| 692 | 46 |
| 863 | 40 |
| 695 | 49 |
| 903 | 26 |
| 907 | 84 |
| 1025 | 96 |
| 1000 | 78 |
| 500 | 103 |
| 540 | 2 |
| 800 | 39 |
| 486 | 107 |
